# Assessing the feasibility and sustainability of fog water harvesting as an alternative water resource

**DOI:** 10.1038/s41598-025-03919-3

**Published:** 2025-06-02

**Authors:** Behzad Ghiasi, Zohreh Hashemi Aslani, Tarkan Alisoltani

**Affiliations:** Faculty of Environment, University of Tehran, Tehran, Iran

**Keywords:** Water supply, Fog water harvesting, Atmospheric water, Cost analysis, Sustainable development, Hydrology, Civil engineering, Freshwater ecology

## Abstract

Iran is experiencing an escalating freshwater crisis due to factors such as population growth, drought, and inadequate water resource management. Atmospheric water, which is six times the volume of all global rivers, presents an untapped potential. This study assesses Iran’s potential for fog water harvesting (FWH) by analyzing atmospheric conditions—including relative humidity, fog density, and frequency—along with technical feasibility and a cost–benefit analysis. Data from 120 synoptic stations identified regions with high fog water potential, notably the southern areas, exhibiting yields up to 65 L/m^2^/day, and northern and eastern regions with potentials between 25 and 45 L/m^2^/day. Compared to similar global projects, Iran’s relative humidity (78%–96%) and the cost of fog water harvesting ($0.25/m^3^) demonstrate superior feasibility over desalination ($0.6/m^3^). Additionally, this study highlights the importance of fog type (e.g., advection vs. upslope) and region-specific limitations that may impact implementation. Sustainability considerations—economic, environmental, and social—are addressed, alongside proposed pilot areas such as Kish and Chabahar. Fog water harvesting is positioned as a promising supplement to Iran’s national water strategy.

## Introduction

Iran’s water scarcity is one of the most pressing environmental challenges it faces, driven by climate change, population growth, and inefficient resource management. Prolonged droughts, such as the devastating 1999–2002 event, underscore the urgency of finding alternative water sources. Despite advancements in desalination and wastewater treatment, these methods remain energy-intensive and cost-prohibitive for remote areas^1,2^. Recent studies indicate that regions with lower mean annual precipitation and elevation experience prolonged droughts. Climate change is expected to intensify Iran’s drought conditions through rising temperatures and reduced precipitation. CMIP6 models project increasing precipitation variability coupled with more severe droughts, particularly under higher emission scenarios^3,4^. While long-term average annual precipitation may increase, heightened variability will likely lead to more intense and prolonged droughts, especially in central and eastern Iran^1^. Major cities such as Tehran, Mashhad, and Isfahan are projected to face more severe meteorological droughts, and reduced runoff in semi-arid regions like the Zard River Basin is expected to exacerbate hydrological drought conditions^5,6^. These droughts could significantly affect drinking water resources in arid and semi-arid regions dependent on surface and groundwater sources, such as springs and reservoirs. To achieve sustainable development, it is essential to optimize water consumption and identify alternative resources to mitigate the risk of a sustainable water supply crisis.

Traditional water sources such as rivers, lakes, groundwater reserves, and dams have long been the primary means of water acquisition. However, due to climate variability and over-extraction, the sustainability of these resources has significantly declined over time^7^. In this context, fog water harvesting offers a complementary approach to supplement traditional sources and reduce the risks associated with water scarcity, particularly in remote and arid regions^8^. This innovative and environmentally friendly technique captures water from atmospheric moisture, providing a sustainable resource for communities, agriculture, and ecosystems.

Fog water harvesting has been practiced since ancient times in arid and semi-arid regions to extract water from atmospheric moisture. Early methods included using rock piles to encourage condensation runoff^9^, placing buckets at the base of natural barriers like plants to collect dripping water, and constructing honeycomb-shaped walls to facilitate mist and dew accumulation near cultivated areas^10^. In recent years, the utilization of this valuable resource has gained renewed attention, highlighting its potential as a reliable and sustainable water source in regions affected by water scarcity. Modern fog water harvesting primarily employs mesh nets, such as polypropylene Raschel mesh, to intercept and collect water droplets, inspired by natural designs like spider webs and desert beetles^11,12^. Recent advancements include Janus membranes with heterogeneous wettability, improving water collection efficiency (WCE) by 736.67%^13–15^ wavy-bumpy yarns enhancing harvesting rates by 700%^14^, cylindrical brush collectors with up to 50% efficiency under high fog speeds^16^, and 3D-printed vertical pillars yielding 3.956 g/h/cm^2^ through edge-effect condensation^17^. These innovations significantly enhance fog water harvesting efficiency and scalability.

Extensive research has demonstrated its feasibility in diverse regions: in Cape Columbine, South Africa, over 2.5 L/m^2^/day was collected, 90% from fog^18^,in Dhofar, Oman, up to 995.04 L/m^2^ over 76 days was harvested^19^,in Ifni, Morocco, inland sites collected 1.7 L/m^2^/day, coastal sites 9.1 L/m^2^/day, with 90% from fog^20^,in Khorasan Razavi, Iran, collectors produced up to 40 L/day during dry periods (40); and in southeast Iran, 6.8 L/m^2^/day was harvested in June, with 20% of atmospheric moisture recoverable^21^. These studies emphasize that fog water harvesting’s potential depends heavily on the spatial and temporal distribution of fog and local environmental conditions. For example, coastal areas and mountainous regions often exhibit the highest fog water collection rates due to favorable topographical and meteorological factors^20,22^. Recent studies highlight that fog water harvesting offers significant advantages, including minimal environmental impact, low cost, ease of maintenance, and scalability, making it particularly suitable for low-income or remote areas^9,17,23^. However, its effectiveness is geographically limited to regions with consistent fog patterns, and the collected water may not suffice for larger populations or industries (Guo et al., 2024). Additionally, challenges such as the need for community involvement, adequate funding, and supportive policies can hinder long-term sustainability^24^. Thus, fog water harvesting is best utilized as a complementary approach alongside other water sources to ensure reliable and sustainable water access.

Despite its historical background and the introduction of innovative methods and numerous studies on fog water harvesting, this approach has not been widely adopted in Iran. Considering the numerous advantages of this method, it is essential to comprehensively assess the potential and feasibility of utilizing this valuable resource in the country. Furthermore, identifying regions with the highest potential for fog water harvesting is crucial. Given the current status of water resources in Iran and the cost of potable water production, an economic analysis is also necessary. Such an analysis would enable the introduction of a new water source, contributing to sustainable development. Regarding this, the potential of extractable water from fog for the entire Iran was investigated using 120 synoptic stations of the Meteorological Organization. A more detailed assessment was conducted to evaluate locations with high potential better. By analyzing the climatic data and fog patterns in the selected locations, the researchers aim to determine the feasibility and potential of fog water harvesting as a viable solution to address the water scarcity challenges faced by rural communities in Iran. Fog water harvesting may involve assessing the technical requirements and costs of implementing such systems and the overall economic and social benefits. The findings of this feasibility assessment could provide valuable insights for policymakers, urban planners, and rural development organizations in Iran. The successful implementation of fog water harvesting projects in the country’s dry coastal regions could improve water security, support agricultural activities, and enhance the quality of life for the affected communities.

Fog water harvesting offers a practical, low-cost alternative, especially in regions with frequent fog events. Modern advancements—such as Janus membranes and 3D-printed collectors—have enhanced the efficiency of fog water harvesting systems, with water collection rates improving by up to 736%. While this technique has been successfully implemented in countries like Chile and South Africa, it remains underutilized in Iran.

Despite advancements in fog water harvesting technologies, their applicability in Iran’s diverse climatic zones has not been systematically analyzed, particularly concerning economic feasibility and practical implementation strategies. This study aims to address this gap by evaluating Iran’s potential for fog water harvesting. Key objectives include: Identifying regions with high fog water potential, assessing the feasibility of implementing fog water harvesting systems, conducting a cost–benefit analysis to compare fog water harvesting with other water production methods, providing practical recommendations for integrating fog water harvesting into national water management strategies.

## Methodology

**Fig. 1:**
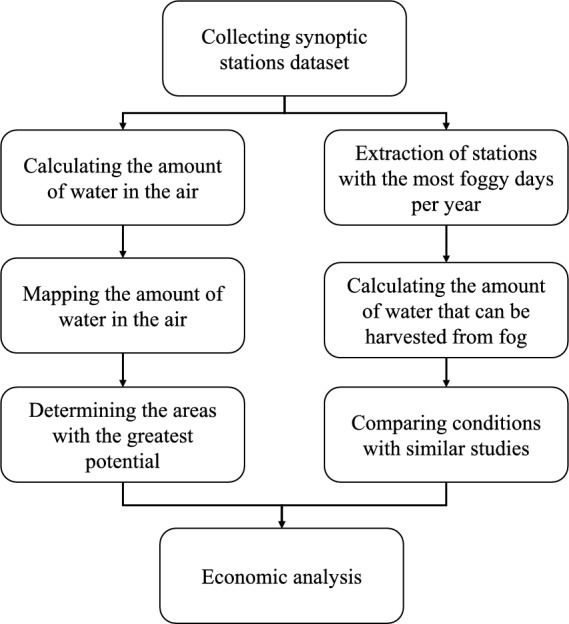
Flowchart of the steps for conducting this research.

The flowchart of this study is illustrated in Fig. 1. Initially, all synoptic data from stations across Iran were collected. These data were utilized in two primary ways: first, to calculate the amount of water available in the atmosphere, as detailed in the subsequent methodology section; and second, to generate a map of atmospheric water potential across the country. Based on this analysis, regions with the highest water harvesting potential were identified. Synoptic station records were then examined to identify locations with the highest number of foggy days annually, enabling comparisons with similar studies conducted in other regions. Finally, an economic analysis was conducted to evaluate the feasibility of fog water harvesting in Iran^25^.

**Fig. 2:**
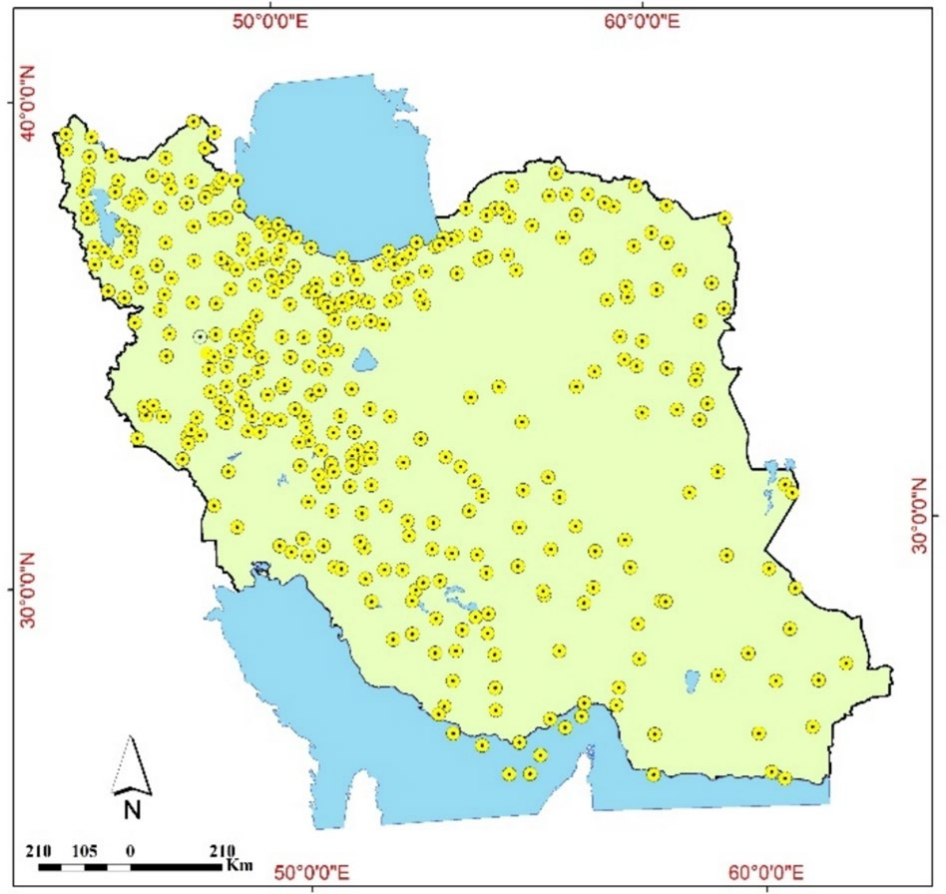
Synoptic stations across the country generated with QGIS 3.34 (www.qgis.org).

This study utilized data from 120 synoptic stations across Iran to evaluate the potential atmospheric water (Fig. 2).

At these stations, the amount of harvestable water from atmospheric humidity was calculated. Atmospheric moisture can be harvested through two mechanisms. The first mechanism involves fog, where larger water droplets can be captured by placing a physical barrier in the path of the fog. The second mechanism is through dew or haze, which requires cooling a surface in contact with humid air to facilitate the formation of larger water droplets that can then be collected.

Numerous factors influence the quantity of water harvested from fog, including wind patterns, mountainous topography, elevation, topographic direction, distance from the coastline, and the specific placement of fog collectors. Therefore, based on the Iran Meteorological Organization dataset, the average temperature, average water vapor pressure, average relative humidity, wind direction, speed, and the number of days with fog and haze at these stations were extracted. The first step in determining the water in air potential involves calculating the absolute humidity using the following equation (6)^26^:

1 $$AH = \frac{216.98}{T} \times e$$

AH is the absolute humidity in grams per cubic meter, T is the air temperature in Kelvin, and e is the vapor pressure at the millibar station. The harvestable water content $Water Collected$ in liters per day per square meter can be calculated using the following equation (6):

2 $$Water Collected = AH \times U \times A \times t \times E$$

Where:

AH represents the absolute humidity in grams per cubic meter.

 U denotes the wind speed in meters per second.

 A is the surface area in square meters.

t signifies the time in seconds.

E is the efficiency of the water harvesting system, which is the percentage of conversion of the available atmospheric water into liquid water. Based on previous studies, the value of E is set to 30% in this research.

Subsequently, the harvestable water content from atmospheric humidity was calculated for the entire country, and a corresponding map was created. This analysis identified regions with higher potential for water harvesting. However, such water collection is only feasible in areas where dense or light fog occurs. The Kish Island, Chabahar, Masouleh, Bandar Kiashahr, and Kouhin stations were selected for further analysis after reviewing meteorological station records regarding the frequency of fog and haze occurrences and ensuring proper distribution of these monitoring stations nationwide.

Meteorological data collection often involves measuring wind speeds at various stations. These measurements are typically recorded in eight distinct directional components, comprising four primary and four secondary directions. In this fog extraction system, the most effective mode is to use a plane perpendicular to the fog’s movement and to use it permanently, so it is necessary to determine the maximum amount of water that can be extracted in different directions before placing the plane perpendicular to that direction^27^. Consequently, the values of collectible water in the eight measured directions are presented in Table 1.

**Table 1:** Wind speed directions.

D8	D7	D6	D5	D4	D3	D2	D1	Title
Northwest	west	Southwest	South	Southeast	East	Northeast	North	Direction
292.5° to 337.5°	247.5° to 292.5°	202.5° to 247.5°	157.5° to 202.5°	112.5° to 157.5°	67.5° to 112.5°	22.5° to 67.5°	337.5° to 22.5°	Angle

### Economic feasibility

To assess the economic feasibility of fog water harvesting, the following costs were analyzed:

#### Capital investment costs

This includes the procurement and installation of fog nets, supporting structures, and auxiliary systems such as storage tanks. Based on previous studies^28^, the initial investment varies between $5 and $830 per square meter, depending on the type of mesh used.

#### Maintenance and operational costs

Unlike energy-intensive technologies such as desalination, fog harvesting requires minimal maintenance, primarily involving periodic cleaning, net replacement (every 10–20 years, depending on material durability), and routine inspections.

#### Water production costs

The cost per cubic meter of fog-harvested water was compared with other conventional methods (Fog Water Harvesting: 0.25 $/m^3^, Municipal Water (Iran): 0.15 $/m^3^ and Desalination: 0.6 $/m^3^).

While fog water harvesting is more expensive than municipal water, it remains a cost-effective alternative in regions where conventional water supply is unavailable or costly. Furthermore, optimizing net materials and selecting suitable harvesting locations can further reduce costs.

## Results and discussion

**Fig. 3:**
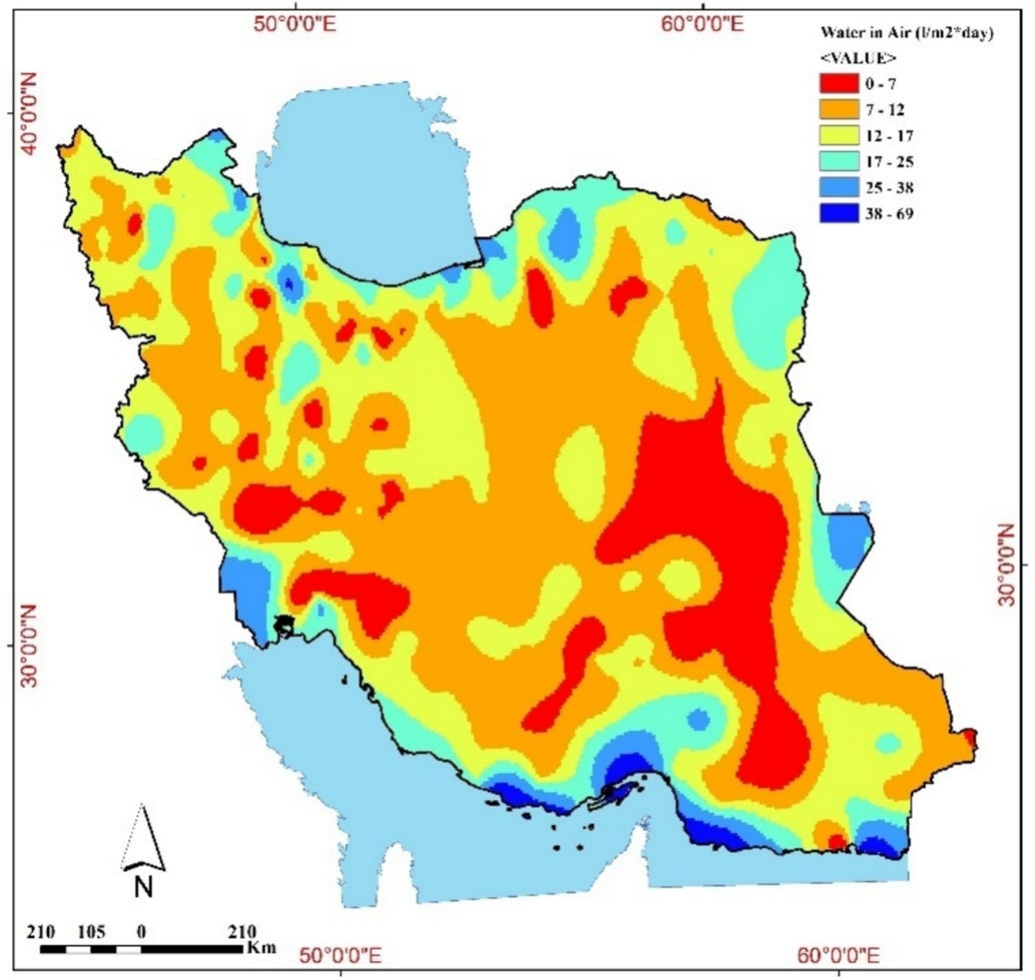
Map of potential atmospheric water availability for Iran generated with QGIS 3.34 (www.qgis.org).

The potential map for harvestable water from fog in Iran was calculated and plotted, as shown in Fig. 3. In addition to relative humidity, parameters such as fog density, droplet size distribution, and fog duration significantly influence the efficiency of fog water harvesting systems. For instance, dense fogs with larger droplets are more effectively intercepted by mesh-based collectors^29^. Moreover, the type of fog plays a critical role,advection fogs, typically found in coastal regions, tend to be more persistent and uniform, providing better harvesting opportunities than upslope (orographic) fogs, which are often localized and intermittent^30^. Incorporating such distinctions can enhance site selection and system design for fog water collection projects. Figure 3 shows that the southern regions, some parts of the country’s north, west, and east have the highest amount of atmospheric water. In the southern regions, up to 65 L of water per square meter per day is available in the air. This amount reaches 34–45 L per square meter per day in some northern regions with high humidity. In some parts of the east and west of the country, approximately 25–34 L of water per square meter per day is available in the air.

In other places, this quantity is negligible and can be essentially ignored. Subsequently, the necessary calculations for the selected stations have been carried out. The higher water availability in southern regions such as Kish is attributable to persistent humidity levels driven by proximity to coastal areas and favorable wind patterns. However, the lower number of foggy days limits practical collection without advanced cooling systems. The collectible water quantities for five selected stations, considering the optimal wind speed directions and a 30% collection efficiency, are calculated and presented in Table 2. Table 2 summarizes the annual fog water collection potential at the selected stations, revealing significant variability. Kish, despite its high atmospheric water content, has fewer foggy days, requiring air cooling systems to enhance water collection. In contrast, Masouleh benefits from consistent fog, achieving efficient collection with basic systems. These findings underscore the importance of tailoring fog water harvesting strategies to local conditions. The number of days with haze and fog at these five stations is further detailed in Table 3. Fog frequency—measured by the number of foggy days per year—was a key parameter in evaluating each location’s practical potential. For example, while Kish exhibits high atmospheric water content, it only experiences 23 foggy days annually, limiting collection without preprocessing techniques. In contrast, Masouleh, with 177 foggy days, demonstrates higher efficiency under basic collection systems. Thus, fog frequency serves as a strong indicator for system viability^31^.

**Table 2:** Collectible fog water at 5 selected stations (L/Day.m2).

Kish	Masouleh	Chabahar	Kouhin	Kiasar	Station
D8	D1	D7	D8	D6	Month/Optimal wind direction
7.4	3.1	4.6	2.3	2.0	January
7.4	1.9	6.1	2.8	2.5	February
9.5	2.3	6.5	3.7	2.7	March
11.9	2.7	8.8	3.3	3.3	April
14.9	4.3	7.2	5.5	4.9	May
14.2	5.3	11.1	6.3	8.2	June
10.4	5.9	7.6	9.3	9.2	July
10.2	7.4	7.0	7.8	6.9	August
13.7	5.0	8.8	5.8	7.4	September
10.2	4.1	5.9	5.1	5.1	October
7.8	2.2	5.5	2.6	2.7	November
7.4	1.2	2.7	2.6	1.6	December
10.4	3.8	7.0	4.6	4.7	Annual average

**Table 3:** Number of days with haze and fog at selected stations.

Kish	Masouleh	Chabahar	Kohein	Kiasar	Station
23	177	0	124	148	Number of days with fog
329	19	221	76	92	Number of days with haze

From a practical standpoint, only fog is considered reliably collectible. Haze can only be converted into water when its air temperature drops, forming larger droplets. Therefore, despite the high potential for water vapor in the air at stations like Kish and Chabahar, these locations typically exhibit a lower overall water collectability. To address this challenge, one must either implement air cooling or condensation techniques at these stations or identify nearby locations at higher altitudes. Considering these factors, the annual collectible water amounts for the selected stations were recalculated and presented in Table 4.

**Table 4:** Average annual collectible water amounts under normal conditions and with air cooling or condensation operations (Liters per Square Meter).

Kish	Masouleh	Chabahar	Kohein	Kiasar	Station
239.2	672.6	0	570	695.6	Normal Condition
3421.6	72.2	1547	349.6	432.4	With Prerequisite Operations

A significant amount of collectible water can be obtained and ultimately utilized by implementing preprocessing operations or choosing higher altitude locations near the stations (Table 4). While Masouleh demonstrates higher annual fog water collection potential under normal conditions, stations like Chabahar require preprocessing techniques to achieve optimal yields. This highlights the need for localized solutions tailored to regional climatic conditions.

Many countries have invested in fog water harvesting initiatives to address various water resource needs. A summary of some of the implemented fog water harvesting projects is provided in Table 5. Comparing the country’s regions’ high potential for fog water harvesting with global studies and projects can assess their global potential. For this purpose, the climatic conditions were examined, focusing on the crucial parameter of relative humidity and the amount of collectible water. Two relevant studies are considered—one conducted in Saudi Arabia by Algarni^32^ and another in the Chabahar region of Iran by Mahmoudi et al.^21^—under the conditions of stations with the lowest potential for collectible water. The results of this comparative analysis are presented in Table 6. The results from Table 6 indicate that the Masouleh station, despite having the lowest potential for water content in the air among the selected stations in Iran, has significantly better relative humidity than the two referenced projects. Considering the statistics in Table 5, Iran presents a better opportunity for practical fog water harvesting due to favorable climatic conditions compared to other locations where efforts have been made due to lower air water content.

**Table 5:** Summary of fog water harvesting projects in various countries.

Description	Country	Row
Providing freshwater for 100 families	Chile	1
Providing water for gardening and visitors to the church	Chile	2
Used for deforestation with native trees and for environmental education	Chile	3
A substrate for ecosystem and climate research. Production rate of 6 lit/m2.day	Chile	4
Providing water for deforestation and restoration of degraded coastal ecosystems, for fruit growth, and for supplying freshwater to the population	Peru	5
Production rate of 12 lit/m2.day for mountainous areas	Ecuador	6
35 collectors with an average production of 6300 L per day in the 4–6 month dry season	Guatemala	7
Production rate of 4000 lit/day	Dominican Republic	8
Production rates between 1 lit/m2.day to 5 lit/m2.day, higher at over 1700 m elevation up to 10 lit/m2.day. They are providing water for schools and domestic use	South Africa	9
Increasing access to drinking water for schools and 120 families	Eritrea	10
Up to 30 lit/m2.day—fog only available for 2 months per year	Oman	11
Average production rate of 30 lit/m2.day for the 3-month winter dry season	Yemen	12
Production rate of 4 lit/m2.day during the summer dry season	Croatia	13
Up to 7 lit/m2.day production rate	Spain	14

**Table 6:** Comparison of relative humidity and water production in two studies with the relative humidity of the city of Masouleh.

Saudi Arabia		Chabahar		Masouleh		
Relative humidity (%)	Fog water harvested (L/Day.m2)	Relative humidity (%)	Fog water harvested (L/Day.m2)	Relative humidity (%)	Potential water harvested (L/Day.m2)	Month/Location
88.0	3.6	61.0	0.3	78.0	3.1	January
93.1	7.8	65.0	1.2	84.0	1.9	February
96.8	1.9	72.0	8.3	93.0	2.3	March
95.4	7.8	73.0	6.5	87.0	2.7	April
90.2	5.7	76.0	0.7	89.0	4.3	May
86.4	1.6	80.0	9.7	80.0	5.3	June
82.1	3.5	81.0	8.7	88.0	5.9	July
77.5	6.4	81.0	6.7	87.0	7.4	August
68.5	3.2	81.0	6.7	96.0	5.0	September
61.5	5.1	77.0	2.6	94.0	4.1	October
75.8	6.2	71.0	4.5	73.0	2.2	November
80.4	9.3	65.0	2.3	84.0	1.2	December
83.0	6.5	73.6	6.5	86.1	3.8	Annual Average

Figure 3 highlights the stark regional disparities in water availability, which align with wind patterns and topographical influences. This demonstrates the critical role of site selection in optimizing fog water harvesting efficiency. Similarly, Table 5 shows that while Kish and Chabahar have high potential, preprocessing techniques like cooling are essential to maximize their yield.

According to recent studies and projects, the cost of harvesting water from fog, including water production and distribution, and assuming volunteer labor, is approximately $1.4 per cubic meter ^28^. This cost increases if a fixed labor force or storage reservoir is required. In six cities in northern Chile, the cost of water production per cubic meter ranges from $1.06 to $3.03, and in the African country of Eritrea, it ranges from $0.71 to $3.3 per cubic meter^28^.

Comparing the cost of fog water production is of great importance. However, the reality is that such costs cannot be directly compared with other water sources, such as desalination, due to differences in the scale of the two technologies. Additionally, fog water harvesting technologies are initially costly to install but require very little maintenance or additional resources afterward. In contrast, technologies like desalination and wastewater treatment demand continuous energy input, chemicals, and labor ^28^.

Table 7 presents the approximate cost per square meter for two different mesh materials used in fog water harvesting worldwide to estimate the cost of fog water production at the Chabahar station. The figures in this table are obtained from the study by Qadir et al. ^28^.

**Table 7:** Comparison of the cost of using different meshes for fog water production at the Chabahar station.

High efficiency 3D compressed networks	Raschel mesh type	Title
830	5 to 50	Cost per square meter of mesh (USD)
2 to 3 times higher	Normal	Water harvesting condition
More than 20 years	10 years	Useful life
80–120	20	Average water production over useful life (cubic meters)
7—10	0.25 – 2.5	Ratio of cost to water produced (USD)

The results of Table 7 indicate that the approximate total cost of fog water harvesting at the Chabahar station, in the most economical scenario, amounts to 0.25 USD per cubic meter. The total cost of conventional water production in Iran is 0.15 USD per cubic meter, and desalination costs 0.6 USD per cubic meter (Iran Ministry of Energy). Additionally, water transportation costs to remote areas should be considered, which can be significant. Moreover, localizing this fog water harvesting technology in the country can significantly reduce the overall cost. As shown in Table 7, fog water harvesting costs vary depending on mesh materials. High-efficiency 3D compressed networks, though costlier upfront, offer greater water yields and longer lifespans, making them suitable for high-potential areas like Kish. The cost-effectiveness of fog water harvesting, combined with its low environmental impact, makes it a viable alternative for rural water supply in Iran.

In comparison to international projects, Iran’s fog collection rates are competitive, with higher relative humidity (78%–96%) than in countries like Chile and Saudi Arabia (65%–85%). Moreover, the cost analysis confirms that fog water harvesting ($0.25/m^3^) is more economical than desalination ($0.6/m^3^) and can supplement conventional water sources in remote regions.

### Challenges and Limitations of FWH in Iran

While fog water harvesting demonstrates promising technical and economic feasibility in various Iranian regions, several challenges may hinder its widespread adoption. First, the spatial limitation of fog occurrence restricts the applicability of this method to specific coastal and high-altitude zones. Additionally, the lack of localized manufacturing capacity for high-efficiency mesh materials could elevate initial costs. Infrastructure development, including storage and distribution networks, may also prove difficult in remote and underdeveloped regions. From a socio-economic perspective, fog harvesting currently suffers from limited public awareness and weak cultural acceptance. Unlike conventional water sources, it is not yet perceived as a reliable or trustworthy option among communities. This skepticism is further reinforced by the relatively low cost of municipal water in Iran, which reduces the economic incentive for both local authorities and national policymakers to invest in alternative methods such as fog harvesting^33^. Furthermore, the prevailing policy approach in Iran continues to prioritize large-scale inter-basin water transfer projects. This orientation toward centralized and capital-intensive solutions poses a significant barrier to the acceptance of decentralized, low-impact innovations like fog harvesting. Overcoming these limitations will require not only technical and financial planning but also robust public education campaigns and a shift in institutional thinking. Without increased stakeholder engagement and government support, the transition from pilot projects to scalable, long-term fog water harvesting systems will remain a challenge.

### Sustainability assessment

The sustainability of this method was evaluated from three perspectives: economic, social, and environmental.

#### Economic sustainability

 As demonstrated in the cost analysis, fog water harvesting requires an initial investment but has negligible operational expenses. Due to its independence from energy-intensive processes, it offers long-term cost advantages. Successful implementation in countries such as Chile, Peru, and Oman highlight its viability for Iran.

#### Social sustainability

In water-scarce regions, fog harvesting can provide reliable water sources for drinking and agriculture, reducing forced migration due to water shortages. Community involvement in the installation and maintenance of fog nets lowers costs and enhances local acceptance. Given its simple design, rural populations can readily adopt and maintain the technology.

#### Environmental sustainability

Unlike desalination, which generates brine waste and consumes significant energy, fog harvesting has negligible environmental impact. By reducing reliance on groundwater extraction, it mitigates land subsidence and groundwater depletion. Regions with frequent fog events, such as Masouleh and Kouhin, provide ideal conditions for long-term sustainability.

## Conclusion

Fog water harvesting emerges as a promising strategy to address water scarcity in Iran, particularly in remote areas lacking access to conventional water networks. This cost-effective and sustainable technique captures atmospheric moisture using specialized mesh structures, with performance influenced by multiple factors including fog frequency, density, droplet size, and type. The study evaluated Iran’s fog harvesting potential using data from 120 synoptic stations, identifying high-yield regions like Kish, Chabahar, Masouleh, and Kouhin. While areas such as Masouleh benefit from frequent fog events under natural conditions, other regions may require preprocessing systems (e.g., cooling) to maximize yield. In addition to technical and economic assessments, the study outlines key challenges including limited spatial applicability, infrastructure needs, and the necessity of local engagement. Still, Iran’s superior relative humidity and competitive costs reinforce the feasibility of FWH compared to global benchmarks. Integrating fog harvesting into national water plans—supported by local manufacturing and pilot programs—could significantly enhance water resilience and contribute to sustainable development goals. Cost analysis demonstrated that fog water harvesting in Iran ($0.25/m^3^) is significantly cheaper than desalination ($0.6/m^3^) and, in some cases, more economical than conventional methods.​

Ensuring the sustainability of fog harvesting projects necessitates active community involvement, securing economic viability, minimizing environmental impacts, and adapting technologies to local conditions. Implementing pilot projects in high-potential areas and integrating fog harvesting into national water management strategies could further optimize its impact, contributing to sustainable development in Iran.

### Key findings include

1. High atmospheric water availability in southern and northern regions, with potential yields of up to 65 L/m^2^/day.
2. Economic feasibility, with costs as low as $0.25/m^3^, outperforming desalination in remote areas.​
3. Superior climatic conditions compared to global projects, enhancing Iran’s suitability for fog water harvesting.​.
4. The necessity of integrating sustainability considerations, such as community engagement and environmental impact, into project planning and implementation.​

​

### Practical recommendations

- Initiate pilot projects in high-potential areas like Kish and Chabahar.​
- Promote localized manufacturing of fog water harvesting materials to reduce costs further.​
- Integrate fog water harvesting into national water management plans to ensure sustainability.​

### Recommendations for future research

- Investigate region-specific mesh materials to enhance efficiency and reduce costs.​
- Explore integration with renewable energy systems for preprocessing techniques.​
- Conduct pilot projects in high-potential areas to assess practical implementation and scalability.​

Fog water harvesting represents a transformative solution for Iran’s water scarcity challenges. By leveraging this untapped resource and addressing sustainability considerations, the country can enhance water security, support agriculture, and achieve sustainable development goals.

## Data Availability

The datasets used and/or analyzed during the current study available from the corresponding author on reasonable request.
